# The anoikis-related gene signature predicts survival accurately in colon adenocarcinoma

**DOI:** 10.1038/s41598-023-40907-x

**Published:** 2023-08-25

**Authors:** Gunchu Hu, Jian Li, Yi Zeng, Lixin Liu, Zhuowen Yu, Xiaoyan Qi, Kuijie Liu, Hongliang Yao

**Affiliations:** 1 Department of General Surgery, The Second Xiangya Hospital of Central South University, 139 Renmin Road, Changsha, 410011 Hunan China; 2 Department of Orthopedics, the Second Xiangya Hospital of Central South University, Changsha, 410011 China; 3 Orthopedic Biomedical Materials Engineering Laboratory of Hunan Province, Changsha, 410011 China; 4 Hunan Key Laboratory of Tumor Models and Individualized Medicine of Hunan Province, Changsha, 410011 China

**Keywords:** Computational biology and bioinformatics, Biomarkers, Gastroenterology, Risk factors

## Abstract

Colon adenocarcinoma (COAD) is a serious public health problem, the third most common cancer and the second most deadly cancer in the world. About 9.4% of cancer-related deaths in 2020 were due to COAD. Anoikis is a specialized form of programmed cell death that plays an important role in tumor invasion and metastasis. The presence of anti-anoikis factors is associated with tumor aggressiveness and drug resistance. Various bioinformatic methods, such as differential expression analysis, and functional annotation analysis, machine learning, were used in this study. RNA-sequencing and clinical data from COAD patients were obtained from the Gene expression omnibus (GEO) and The Cancer Genome Atlas (TCGA) databases. Construction of a prognostic nomogram for predicting overall survival (OS) using multivariate analysis and Lasso-Cox regression. Immunohistochemistry (IHC) was our method of validating the expression of seven genes that are linked to anoikis in COAD. We identified seven anoikis-related genes as predictors of COAD survival and prognosis, and confirmed their accuracy in predicting colon adenocarcinoma prognosis by KM survival curves and ROC curves. A seven-gene risk score consisting of NAT1, CDC25C, ATP2A3, MMP3, EEF1A2, PBK, and TIMP1 showed strong prognostic value. Meanwhile, we made a nomogram to predict the survival rate of COAD patients. The immune infiltration assay showed T cells. CD4 memory. Rest and macrophages. M0 has a higher proportion in COAD, and 11 genes related to tumor immunity are important. GDSC2-based drug susceptibility analysis showed that 6 out of 198 drugs were significant in COAD. Anoikis-related genes have potential value in predicting the prognosis of COAD and provide clues for developing new therapeutic strategies for COAD. Immune infiltration and drug susceptibility results provide important clues for finding new personalized treatment options for COAD. These findings also suggest possible mechanisms that may affect prognosis. These results are the starting point for planning individualized treatment and managing patient outcomes.

## Introduction

COAD is the fourth most common malignancy with an estimated 247,563 deaths in China in 2018^1^. COAD is a disease that occurs only in the colon and is caused by excessive proliferation of epithelial cells in the colonic gland. There are three main types of COAD: sporadic, hereditary, and colitis-associated. If COAD is detected early enough, it can be completely cured with surgery and treatment. However, high recurrence rates and anticancer drug resistance increase the rate of treatment failure rates^2^. In recent years, with the development of the economy, the incidence and mortality of COAD have risen rapidly^3^. Molecular studies are playing an increasingly important role in predicting prognosis and determining optimal treatment in poor-prognosis colon cancer^4^. Emphasizing the status, development, risk factors and controls of COAD is important to raise public awareness.

Anoikis, a form of programmed cell death resulting from loss of interaction with the cells outer membrane, is known as a physiological metastatic disease^5–7^. Anoikis occurs when cells detach from their normal extracellular matrix (ECM), which provides them with signals for survival and growth. By inducing anoikis, the body prevents cells from growing and implanting in inappropriate locations, such as other organs, where they could cause harm. However, tumor cell metastasis is the process by which cancer cells spread from their primary site to distant sites in the body, forming secondary tumors. To achieve metastasis, tumor cells must overcome anoikis and survive in ECM-deprived conditions. Cancer cells must develop anoikis resistance to survive in the bloodstream before metastasizing to distant organs^8,9^. Acquired anaerobic resistance allows cancer cell survival in circulation and is important for metastatic progression. However, the molecular mechanism of anoikis resistance in tumor cells remains unknown. Jin Lingtao confirmed that the increased expression of GDH1 mediated by the transcription factor PLAG1 and its downstream substrate AMPK can provide anti-anoikis and pro-metastasis signals for lung cancer cells^10^. Lindsay J shows overexpression of CBX2 in high-grade primary serous ovarian cancer tumors and cell lines to prevent anoikis in suspension culture, showing high-grade serous ovarian cancer cells under different culture conditions. Lindsay J show that loss of CBX2 is relevant. It is associated with decreased proliferation, increased blood sensitivity and decreased stem cell numbers^11^. Anoikis-related genes are genes that control cell-ECM communication, which avoid cell proliferation and implantation in inappropriate locations during tissue formation and maintenance. Moreover, they also involve cancer cell anoikis tolerance, invasive ability and TME attributes, which are associated with tumor clinical characteristics, prognosis and efficacy. Therefore, they are candidate biomarkers or targets for tumors.

The corresponding information of the COAD samples was obtained by mining the TCGA database. We then used Genecards to isolate anoikis-related genes. A prediction model of anoikis-related genes in COAD was then constructed and validated using the GEO database. Simultaneously, immune infiltration and drug susceptibility tests were performed. The purpose of this study is to construct prognostic markers related to COAD and investigate their potential mechanism of action and clinical value in COAD and make recommendations to find new treatment options.

## Materials and methods

### Acquisition and processing of data and DEGs identification

Expression sets and clinical information [gender, age, OS, PFS, TNM stage] (expression was measured as raw read counts using STAR, then converted to transcripts per million (TPM). Table 1 listed the baseline clinical characteristics of TCGA-COAD. Only COAD patients with survival information were included in this study. Anoikis-related genes were obtained from Gendcards (https://www.genecards.org/).

**Table 1:** Clinical characteristics of COAD samples from the TCGA database.

Basic characteristics	Variables	Overall (N = 426)
Age (years)	Median (1st Qu, 3rd Qu)	68.00 (58.00, 77.00)
Sex	Male	229 (53.8%)
	Female	197 (46.2%)
History of colon polyps	Yes	127 (29.8%)
	No	234 (54.9%)
	Unknown	65 (15.3%)
History of neoadjuvant treatment	Yes	3 (0.7%)
	No	423 (99.7%)
Histological type	Colon adenocarcinoma	363 (85.2%)
	Colon mucinous adenocarcinoma	59 (13.9%)
	Unknown	4 (0.9%)
TNM stage	Stage I	75 (17.6%)
	Stage II	167 (39.2%)
	Stage III	124 (29.1%)
	Stage IV	60 (14.1%)
T stage	T1	11 (2.6%)
	T2	74 (17.4%)
	T3	292 (68.5%)
	T4	49 (11.5%)
N stage	N0	251 (58.9%)
	N1	101 (23.7%)
	N2	74 (17.4%)
M stage	M0	365 (85.7%)
	M1	61 (14.3%)
Longest dimension(cm)	≤ 1 cm	125 (29.3%)
	> 1 cm	124 (29.1%)
	Unknown	177 (41.6%)
Residual tumor	R0	310 (72.8%)
	R1	4 (0.9%)
	R2	21 (4.9%)
	Rx	91 (21.4%)
Survival status	Alive	334 (78.4%)
	Dead	92 (21.6%)
OS.time (mouths)	Median (1st Qu, 3rd Qu)	13.23 (23.07,36.50)
Progression-free-survival status	No progressive disease	311 (73.0%)
	Progressive disease	115 (27.0%)
PFS.time (mouths)	Median (1st Qu, 3rd Qu)	20.28 (12.22,33.52)

Using the Limma package^12^, a linear model of the microarray data was used to distinguish DEGs from COAD patients and healthy controls in an integrated microarray expression matrix. The false discovery rate (FDR) was examined using the Benjjamini-Hochberg method. A P-value < 0.05, and |log2 (fold change)|(|logFC|) ≥ 1 indicates a significant difference. Heatmaps and volcano maps were created using R. ComplexHeatmap^13,14^ and ggplot2 package.

### Gene enrichment analysis

To investigate possible related pathways of COAD, we performed Gene Enrichment Analysis (GSEA) using the “GOSemSim” package^15,16^, “clusterProfiler” package^17^, “biomaRt” package^18,19^ and “stringi” package in R.GO Enrichment and KEGG pathway analysis. The “ggplot2” package displays the results. Based on the associated anoikis, we used the Genecards online resource to exclude genes that might play a role in anoikis. The screening criteria were as follows: category = “encoded protein” score cut-off > 0.15.

### Colon adenocarcinoma CNVs analysis

Whole exome/genome sequence (WXS/WGS) somatic mutation COAD data were downloaded from the GDC-TCGA-COAD project to the UCSC-Xena server. The Utect2 algorithm assigns high confidence to somatic variants and detects other germline mutations. Tumors were mapped in descending order of mutation using the R package “maftools”^20^.

### Construction of risk score model and validation

A total of 426 samples will be selected from the TCGA-COAD dataset as the training cohort. In the training cohort, 189 anoikis-related genes were screened based on univariate Cox analysis. Thirteen potential prognostic genes were then screened using the Least Absolute Shrinkage Regression and Selection Operator (LASSO). Finally, only 7 genes were included in the risk signature according to the results of the multivariate cox regression. The risk score was calculated according to the gene expression level and the regression coefficient with “glmnet” package^21^, and the formula was risk score = coefficient Σ(Genei) × expression(Genei). According to the principle of median risk score, the OS of this group was divided into high-risk group and low-risk group, and the “Survivor” R package was run for analysis. We then used KM survival curves and ROC curves to evaluate their value in predicting OS survival prognosis with “pROC” package^22^. In addition, risk score histograms, survival status scatterplots, and DEGs distribution heatmaps were generated for both groups of patients. Survival curves from the validation set (GSE39582, 1 ≤ OS.time ≤ 120, mouths) were used to assess the reliability of the validated group risk assessment model. The main clinical features of GSE39582 (1 ≤ OS.time ≤ 120, mouths) are shown in Table 2. The 1-, 3-, and 5-year survival rates of COAD patients were also predicted from the training and validation sets.

**Table 2:** Clinical characteristics of COAD samples from the GSE39582 (1 ≤ OS.time ≤ 120, mouths).

Basic characteristics	Variables	Overall (N = 491)
Age (years)	Median (1st Qu, 3rd Qu)	68.10 (58.00, 76.00)
Sex	Male	268 (54.6%)
	Female	223 (45.4%)
Cin.status	+	311 (63.3%)
	–	102 (20.8%)
	Unknown	78 (15.9%)
Cimp.status	+	83 (16.9%)
	–	354 (72.1%)
	Unknown	54 (11.0%)
TNM stage	Stage I	30 (6.1%)
	Stage II	219 (44.6%)
	Stage III	183 (37.3%)
	Stage IV	59 (12.0%)
T stage	T1	9 (1.8%)
	T2	43 (8.8%)
	T3	332 (67.6%)
	T4	107 (21.8%)
N stage	N0	264 (53.8%)
	N1	124 (25.2%)
	N2	103 (21.0%)
M stage	M0	431 (87.8%)
	M1	60 (12.2%)
Kras.mutation	WT	282 (57.4%)
	M	188 (38.3%)
	Unknown	21 (4.3%)
Tp53.mutation	WT	137 (27.9%)
	M	162 (33.0%)
	Unknown	192 (39.1%)
Braf.mutation	WT	398 (81.1%)
	M	47 (9.6%)
	Unknown	46 (9.3%)
MMR.status	pMMR	389 (79.2%)
	dMMR	69 (14.1%)
	Unknown	33 (6.7%)
Chemotherapy.adjuvant	+	205 (41.8%)
	–	270 (55.0%)
	Unknown	16 (3.2%)
Survival status	Alive	320 (65.2%)
	Dead	171 (34.8%)
OS.time (mouths)	Median (1st Qu, 3rd Qu)	50.00 (26.00,75.00)

### Immune cell infiltration analysis

We extracted expression data from 22 tumor-infiltrating immune cells using the Cybersort platform. We then analyzed this using sample OS data and implemented algorithms using CyberSort to estimate the proportion of different immune cells among OS cells. The algorithm is based on Monte Carlo sampling, providing scattered p-values for each sample.

### Drug sensitivity analysis

The “OncoPredict” R package^23^ was used to search susceptibility data in the GDSC2 database and to predict drug COAD responses in the GDSC2 database. Spearman correlation analysis was then performed to obtain drugs associated with risk outcomes and plot the correlation scatterplot with “ggstatsplot” package.

### Immunohistochemistry for model genes

We performed IHC using clinical samples of COAD to confirm the expression differences of model genes in COAD clinically. For IHC experiments, we collected 70 tissue sections (five samples per COAD and tumor-adjacent normal tissue). We rehydrated the sections with ethanol and dewaxed them with xylene. Then we treated them with 3% H_2_O_2_ for 20 min to block endogenous peroxidase and with 1 mM EDTA to retrieve the antigen. We incubated the sections with 1:50 diluted model genes antibody (From: SANTA CRUZ BIOTECHNOLOGY) at 4 °C overnight. After incubation with a 2-step plus PolyHRP Anti-Mouse/Rabbit IgG Detection System (PV-9000, Zhongshan Jinqiao Biotechnology Company, Beijing, China), we visualized the sections with diaminobenzidine (DAB; Zhongshan Jinqiao Biotechnology Company, Beijing, China), counterstained them with hematoxylin, and dehydrated them. We scanned the sections using a Zeiss microscope. We measured and quantified staining intensity using the ImageJ plug-in “IHC Toolbox” and GraphPad Prism version 7 software, respectively. We considered p-value < 0.05 as significant.

### Statistical analysis

In this experiment, all statistical analyzes were performed using R 4.2.2. The Wilcoxon test was used to compare nonparametric data from two independent samples. Parametric data were analyzed by *t* test and one-way ANOVA. A P value <  0.05 was considered statistically significant (*p-value < 0.05; **p-value < 0.01; ***p-value < 0.001). Related R packages include ggplot2, ggpubr, survival, survminer, and others taken from the Bioconductor package or the R package. P < 0.05 was considered statistically significant for each analysis.

## Results

### Identification of DEGs and GSEA analysis

**Figure 1:**
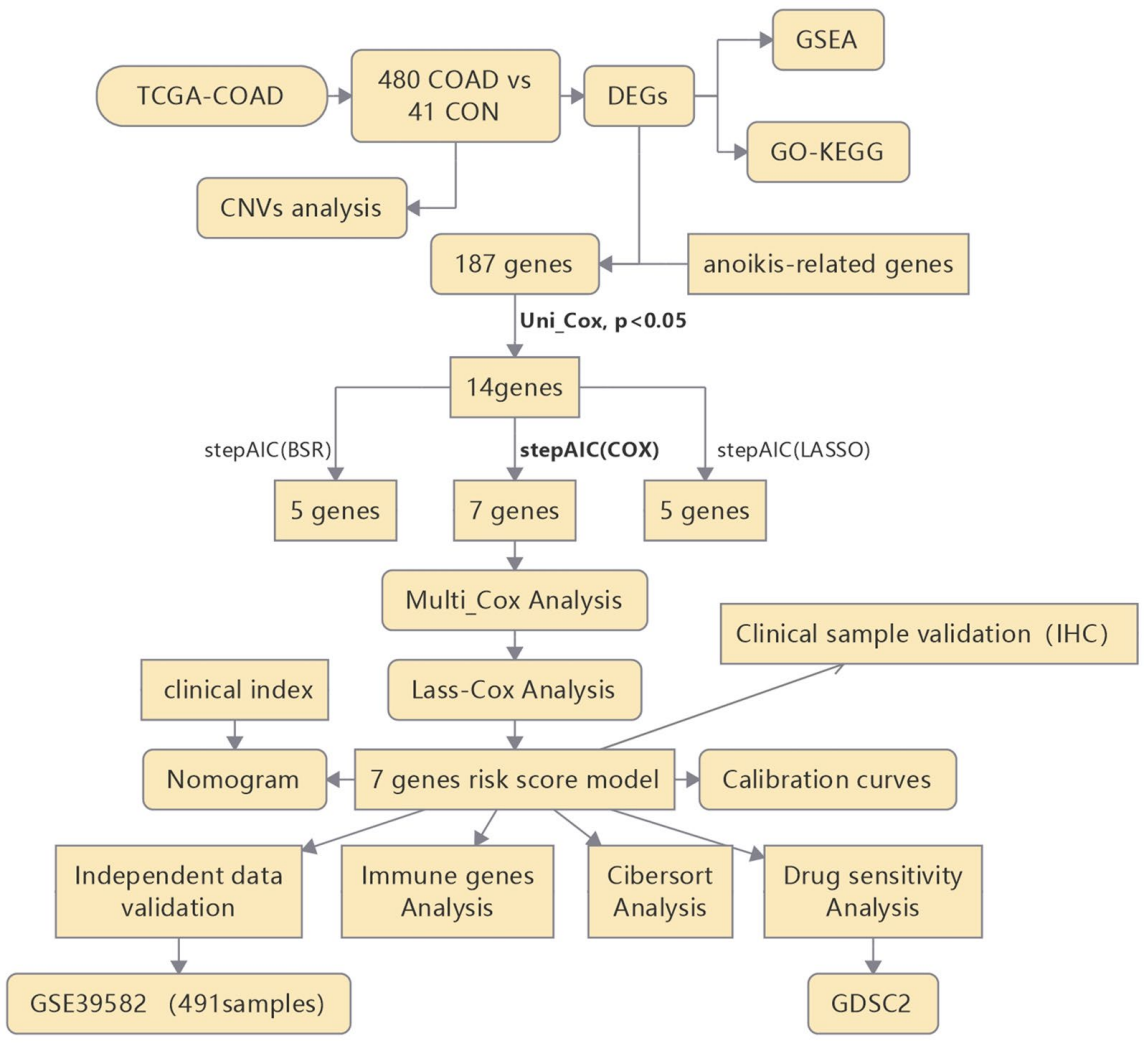
Flow chart of this study.

**Figure 2:**
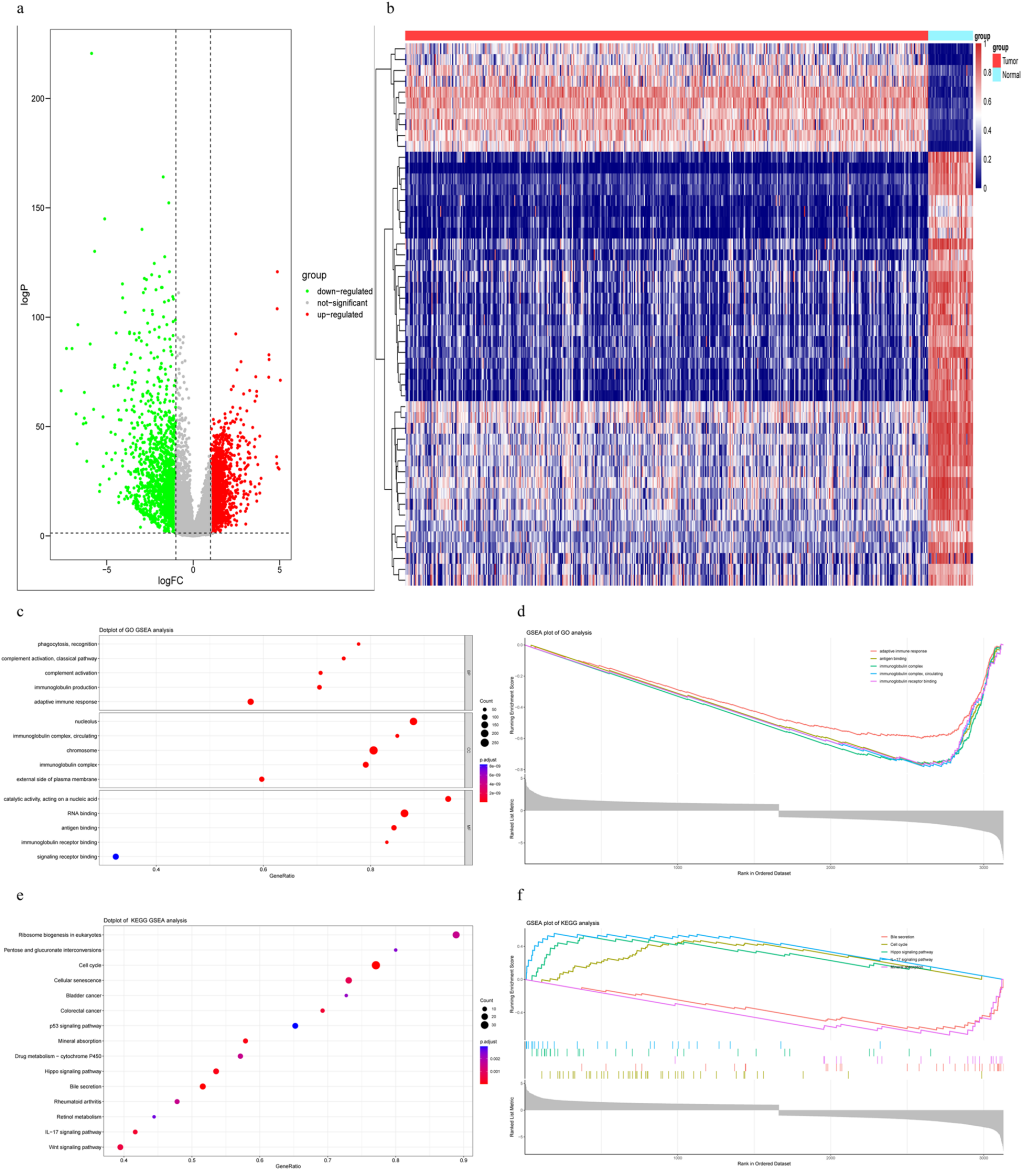
DEGs screening in integrated TCGA database and GSEA analysis. (**a**) Volcano plot of COAD samples from the TCGA database, Data points in red represent up-regulated, and green represent down-regulated genes, (**b**) Heatmap of DEGs identified in integrated microarray. Legend on the top right indicates the change of the genes, (**c**,**d**) GO results of GSEA analysis, (**e**,**f**) KEGG results of GSEA analysis.

Figure 1 is the flow chart of our study. Based on the Limma package and previously defined thresholds, a total of 3335 DEGs that could be further enriched were identified, of which 1547 were downregulated and 1788 were upregulated (Supplementary Table S1). Figure 2a shows the DEG volcano map and Fig. 2b shows the heatmap.

First, GSEA analysis is performed to examine the most relevant genetic ontologies. As shown in Fig. 2c,d (Supplementary Table S2), “adaptive immune response”, “antigen binding”, “immunoglobulin complex” and “immunoglobulin receptor binding” were the most abundant terms in Gene Ontology. GSEA pathway accumulation analysis showed that DEGs were enriched in pathways such as “colon cancer”, “mineral absorption”, “hippo pathway” and “IL-17 pathway” (Fig. 2e,f) (Supplementary Table S3).

### GO and KEGG analysis as well as CNVs analysis

**Figure 3:**
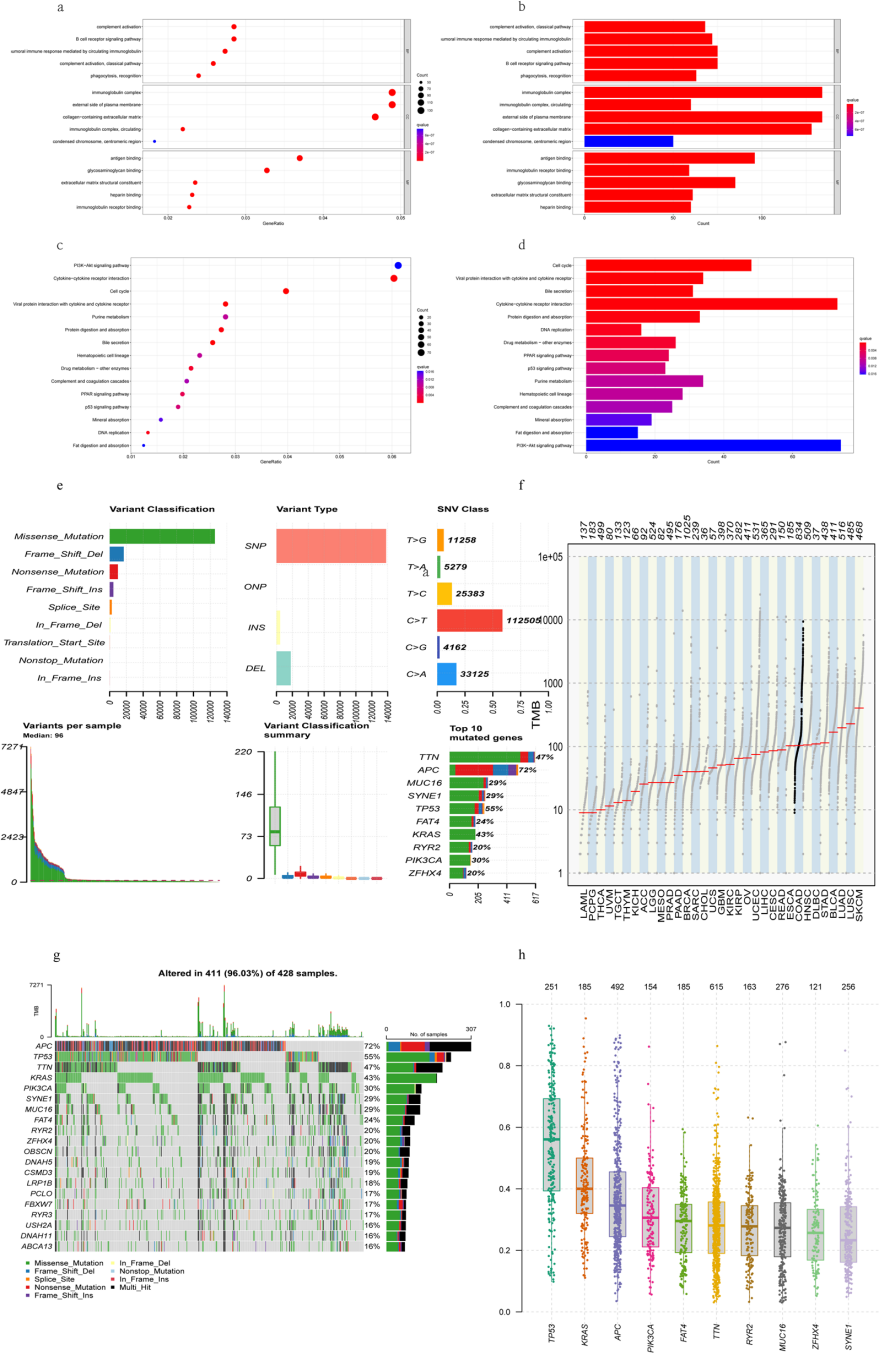
Enrichment Analyses of the DEGs from exprset and CNVs analysis in COAD. (**a**,**b**) Results of GO analysis with DEGs, (**c**,**d**) Results of KEGG analysis with DEGs, (**e**–**h**) the CNV and mutation frequency in COAD. *p < 0.01, ***p < 0.001; *COAD* colon adenocarcinoma, *SNP* single nucleotide polymorphism, *INS* insertion, *DEL* deletion.

GO analysis demonstrated that these 3335 DEGs were primarily enriched in complement activation, B cell receptor signaling pathway, and humoral immune response mediated by circulating immunoglobulin in the biological process, immunoglobulin complex, external side of plasma membrane, and collagen-containing extracellular matrix in the cellular components, antigen binding, glycosaminglycan binding, and extracellular matrix structural constituent in the molecular functions (Fig. 3a,b) (Supplementary Table S4). The KEGG pathway enrichment analysis indicated that these DEGs were enriched in the pathways such as PI3K-Akt signaling pathway, Cytokine-cytokine receptor interaction an Viral protein interaction with cytokine and cytokine receptor (Fig. 3c,d) (Supplementary Table S5).

According to the classification of mutations, we found that missense mutations were the most common (Fig. 3e). SNPs were the most common type of variation, with C > T (112,505) ranking first in the single nucleotide variation (SNV) category. We found a higher tumor mutational burden in COAD (Fig. 3f). We also found that APC (72%) and TP53 (55%) were more frequently mutated than other genes (Fig. 3g). We found the variant allele frequencies for the top 10 genes in COAD (Fig. 3h).

### Filter anoikis-realted genes

We excluded genes that might play a role in anoikis using the online resource Genecards. Screening criteria: category = “encoding protein”, score cutoff > 0.15 (Supplementary Table S6).

**Figure 4:**
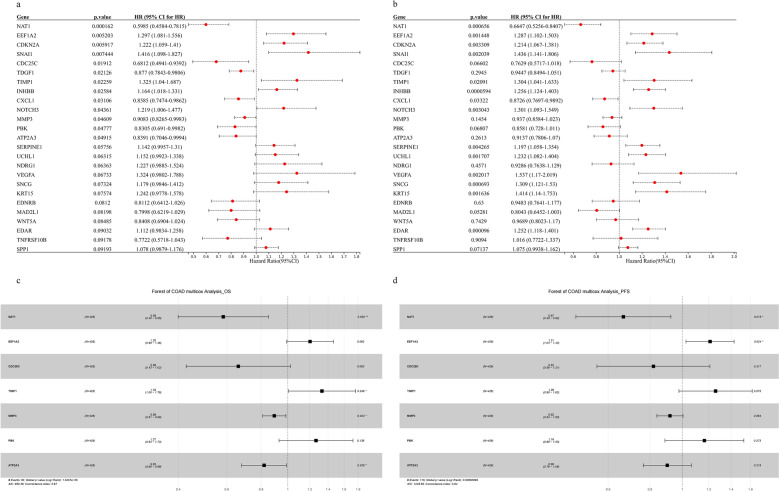
Forest plots for anoikis-related genes from univariate and multivariate cox proportional hazards mode. (**a**) The effect of 25 anoikis-related genes on the overall survival of TCGA datasets, (**b**) The effect of 25 anoikis-related genes on the progress free survival of TCGA datasets, For (**c**) OS and (**d**) PFS, hazard ratios and p-value of the constituents involved in multivariate Cox regression in COAD.

Then, we take the intersection of DEGs and the anoikis-related genes (Supplementary Fig. S1, Table S7). Next, we performed univariate COX hazard analysis on 187 genes, with OS as the outcome measure (Supplementary Table S8), with PFS as the outcome measure (Supplementary Table S9). We selected 25 genes to draw the forest plot of Univariate Cox regression (Fig. 4a,b). Afterwards, we performed stepwise backward regression on the results separately. The variables of the three different models are variables screened by Univariate Cox (p < 0.05), variables screened by All-Subsets Regression (BSR) (Supplementary Fig. S2), and variables screened by LASSO regression. After comparing the three methods (Supplementary Fig. S3), 7 genes were finally selected to draw the forest plot of multicox regression (Fig. 4c,d).

### Establishment of the seven-gene risk signature

**Figure 5:**
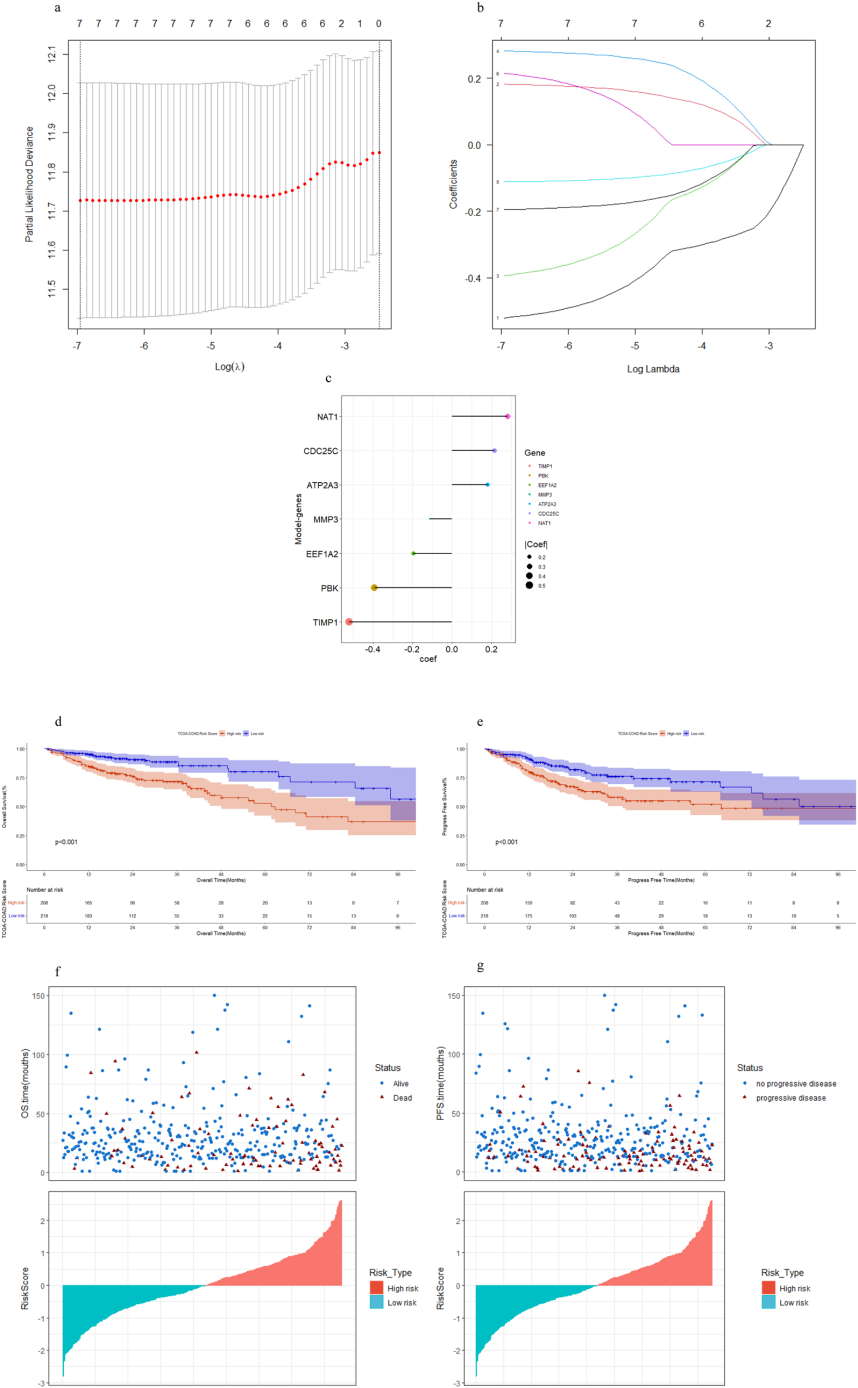
Construction of the risk score. (**a**,**b**) The least absolute shrinkage and selection operator (LASSO) method of anoikis-related genes associated with prognosis, (**c**) The risk score for predicting the survival and prognosis of patients with COAD, (**d**) Kaplan Meier plot of the anoikis signature and overall survival, (**e**) Kaplan Meier plot of the anoikis signature and progress-free survival, (**f**) distribution of risk score, overall survival status, (**g**) distribution of risk score, progress-free survival status.

**Figure 6:**
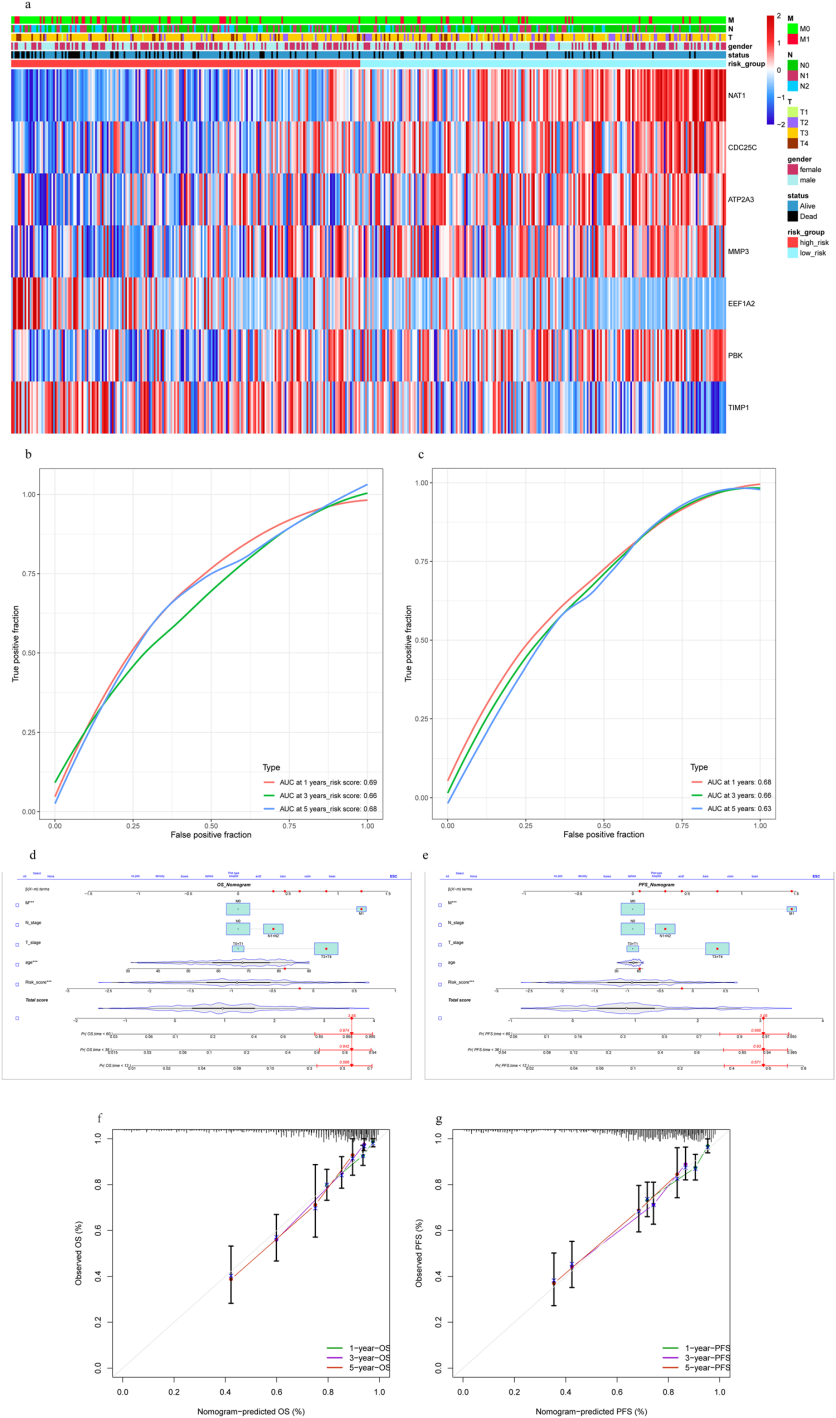
Nomogram development and validation. (**a**) Heatmap of model-genes identified, Legend on the top right indicates the characteristic of the samples, (**b**) ROCs for 1-year, 3-year and 5-year survival prediction, (**c**) ROCs for 1-year, 3-year and 5-year progression-free survival prediction. Nomogram to predict the 1-year, 3-year and 5-year (**d**) OS and (**e**) PFS rate of COAD patients. Calibration curve for the (**f**) OS and (**g**) PFS nomogram model in COAD. A dashed diagonal line represents the ideal nomogram.

We performed Lasso algorithm on these 7 DEGs and regarded them as candidate genes (Fig. 5a,b). Generation of risk scores using seven anoikis-related genes to predict survival and prognosis in COAD patients (Fig. 5c). The risk score = − 0.52021*NAT1 + 0.18218*EEF1A2 + (– 0.39270*CDC25C) + 0.28190*TIMP1 + (− 0.11126*MMP3) + 0.21511*PBK + (− 0.19454*ATP2A3). KM analysis showed that the training set has a high risk score. Corresponded with poorer overall survival (HR = 2.78 (1.97–3.93), log-rank p = 6.45*10^–9^) and poorer progress free survival (HR = 2.13 (1.58–2.88), log-rank p = 7.20*10^–7^) (Figs. 5d,e, Supplementary Table S10). Seven genetic risk signatures could effectively divide patients into a high-risk group and a low-risk group with an intermediate risk score. Therefore, we can intuitively conclude that the patients who died basically happened first were higher ranking (higher risk score) (Fig. 5d–g). We draw the distribution of gene expression in the 426 COAD samples (Fig. 6a). We also applied a risk score covering all relevant genes to estimate OS and PFS after 1, 3, and 5 years (Fig. 6b,c). The prediction accuracy evaluated by AUCs was reported to be 0.69, 0.66 and 0.66 in the 1-year, 3-year and 5-year ROC curves, respectively. The same analyses were conducted for the PFS outcome.

### Nomogram development and validation for COAD

To facilitate the clinical application of the predictive model, we integrated the clinical information and genetic characteristics of TCGA patients, and constructed a nomogram using a multivariate Cox regression model (Fig. 6d,e). Use the discrimination and calibration method to identify and calibrate OS and PFS results. The c-index for OS was 0.775 and the c-index for PFS was 0.742 (Supplementary Table S11), reflecting the superior predictive power of the nomogram. Furthermore, standard curves showed good agreement between predicted and observed OS or PFS between 1, 3, and 5-year survival (Fig. 6f,g).

### Validation of the 7 genes risk score model based on GEO dataset

**Figure 7:**
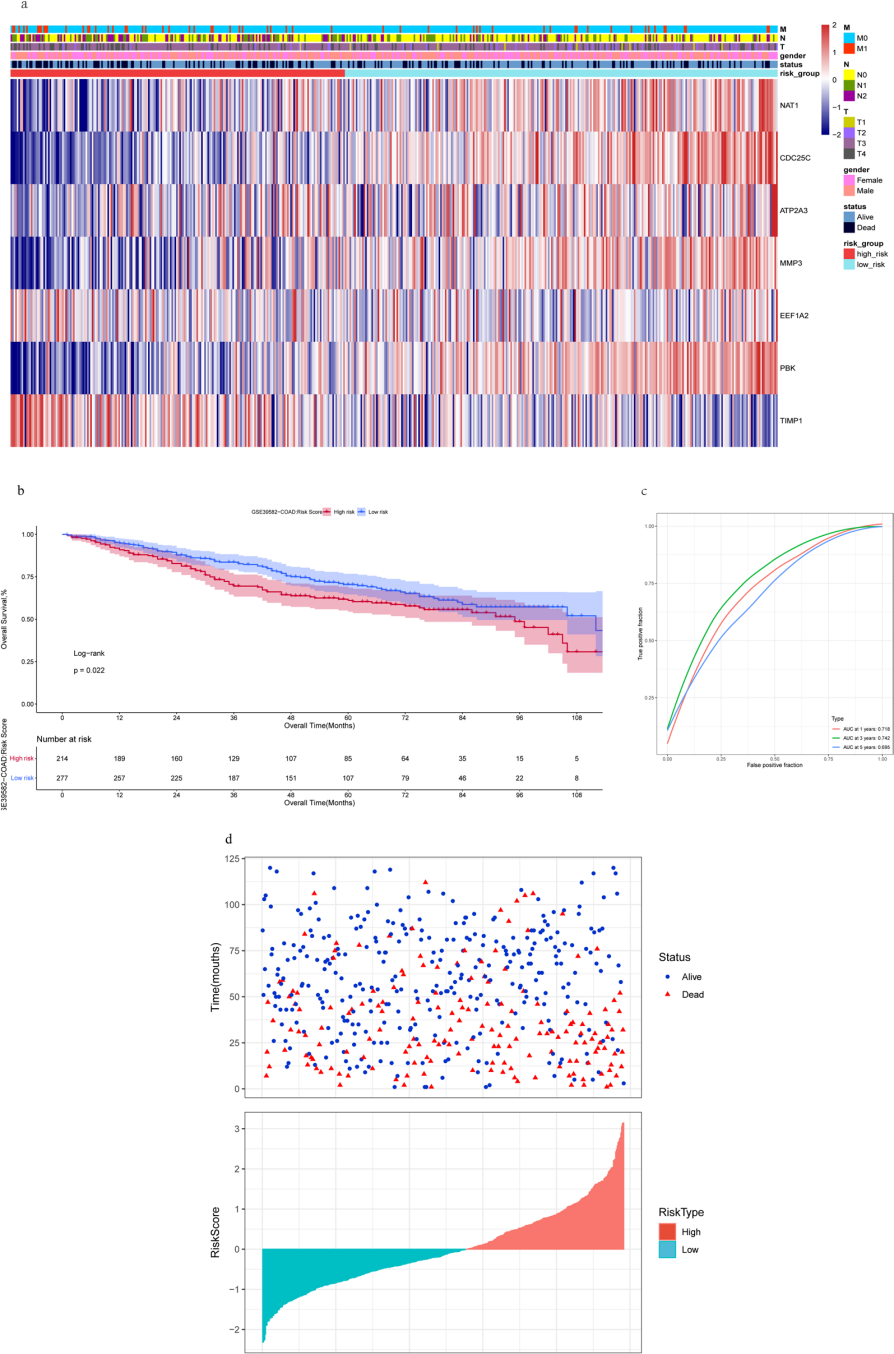
Validation of the seven-gene Signature. (**a**) Heatmap of model-genes identified in validation dataset, Legend on the top right indicates the characteristic of the samples, (**b**) Kaplan Meier plot of the anoikis signature and overall survival in validation dataset. (**c**) ROCs for 1-year, 3-year and 5-year survival prediction in validation dataset. (**d**) Distribution of risk score, overall survival status.

We select GSE39582 (1 ≤ OS.time ≤ 120, mouths) as the validation data set. We plotted a heatmap of the gene expression distribution of the 532 COAD samples (Fig. 7a). KM analyses showed that the validation set has a high risk score corresponded with poorer overall survival (Fig. 7b). The predictive accuracies of AUC results were 0.718, 0.742, and 0.5695 for the 1-year, 3-year, and 5-year ROC curves, respectively (Fig. 7c). We plot scatterplots and histograms to describe the survival status and risk score distributions of these COAD samples (Fig. 7d).

### Immune analysis

**Figure 8:**
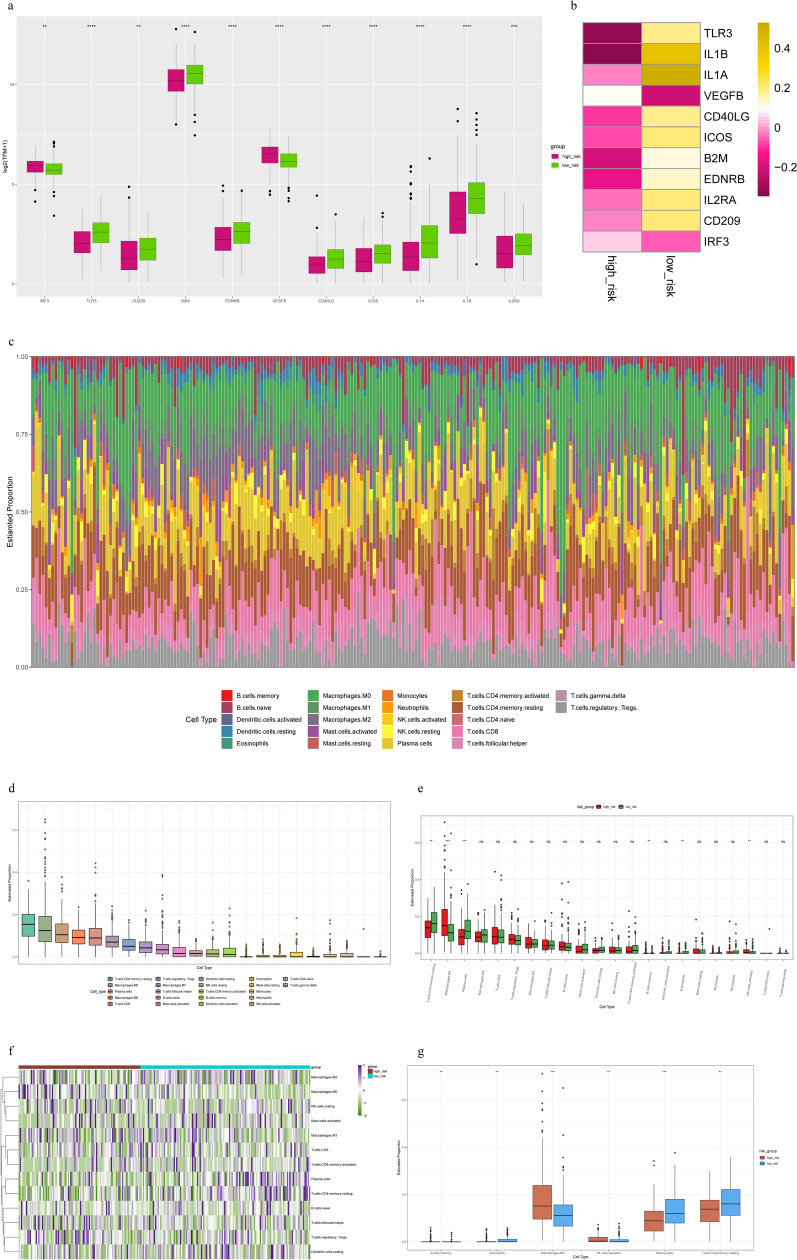
Immune analysis. (**a**,**b**) Comparisons of immune-related genes between the two risk groups in COAD patients. (**c**,**d**) Immune infiltration of 22 dfferent types of immune cells. (**e**) Immune cell component between high-risk group and low-risk group. (**f**) The landscape of immune infiltration in two risk groups for COAD patients. (**g**) Boxplot visualizing differentially immune infiltration. *p < 0.05; **p < 0.01; ***p < 0.001.

We analyzed genes related to the immune system, looked for genes that were differentially expressed and drew boxplots. IRF3 and VEGFB expression increased in the high-risk group and vice versa (Fig. 8a). The distribution of these genes in the two group of COAD samples is shown in Fig. 8b. The occurrence and development of COAD is affected by the tumor microenvironment, and T cells, memory CD4 cells, M0 macrophages, and plasma cells are the most common tumor-infiltrating immune cells (Fig. 8c,d). As a result of the immunological analysis, statistically significant differences in the frequencies of six types of immune cells were observed between the favorable and poor prognosis groups (Fig. 8e–g). It has been suggested that the two groups differed in the ratio of mesenchymal cells to immune cells, possibly resulting in different tumor purities. These results suggest that immune infiltration and immune microenvironment are important for OS in COAD patients.

### Correlation of risk score with drug sensitivity

**Figure 9:**
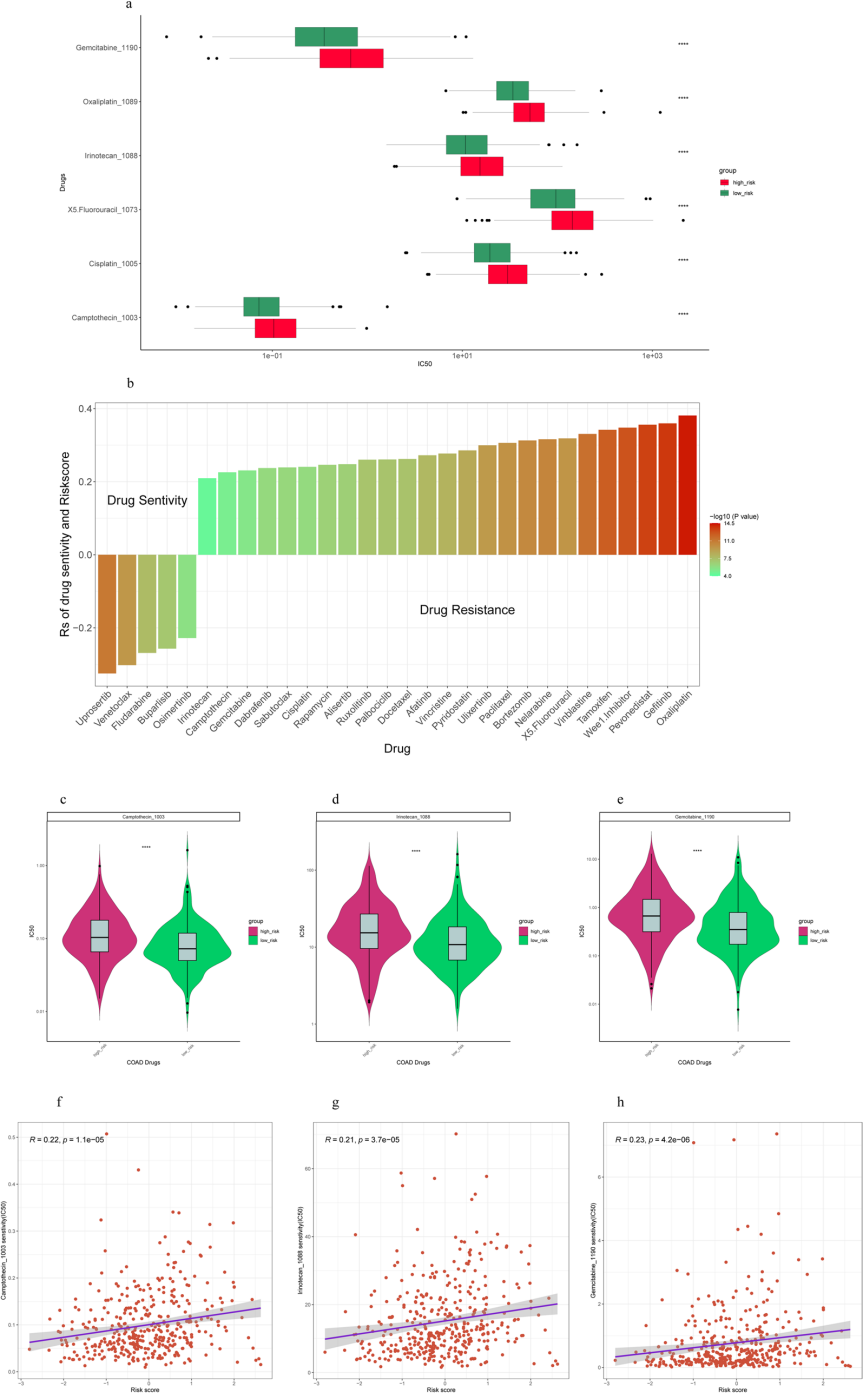
Drug sensitivity. (**a**) Comparisons of 6 COAD chemotherapy drugs on GDSC2 database. (**b**) Assessing drug sensitivity of COAD tumor based on the risk score. (**c**–**e**) Boxplot visualizing differentially drugs. (**f**–**h**) The correlation analysis between risk score and the IC50 of 3 chemotherapy drugs.

We evaluated the value of risk scores in predicting drug sensitivity in different cancer types. We tested 30 drugs in the GDSC2 database whose risk scores were significantly associated with drug sensitivity according to Spearman correlation analysis. The risk score is negatively sensitive to five drugs including Uprosertib, Venetoclax, Fludarabine, Buparlisib, and Osimertinib, and positively correlated with sensitivity to 25 drugs, including Oxaliplatin, 5-Fluorouracil, Cisplatin, Gemcitabine, Camptothecin, Irinotecan and others (Fig. 9a,b,f–h). In conclusion, the establishment of the risk score helps us consider appropriate and effective treatment strategies. The IC50 of three drugs in the high-risk group was lower than low-risk group (Fig. 9c–e). These three drugs are Camptothecin, Irinotecan, Gemcitabine, which indicates that they are expected to become potential drugs for the treatment of COAD.

### Expression of model genes in clinical samples

**Figure 10:**
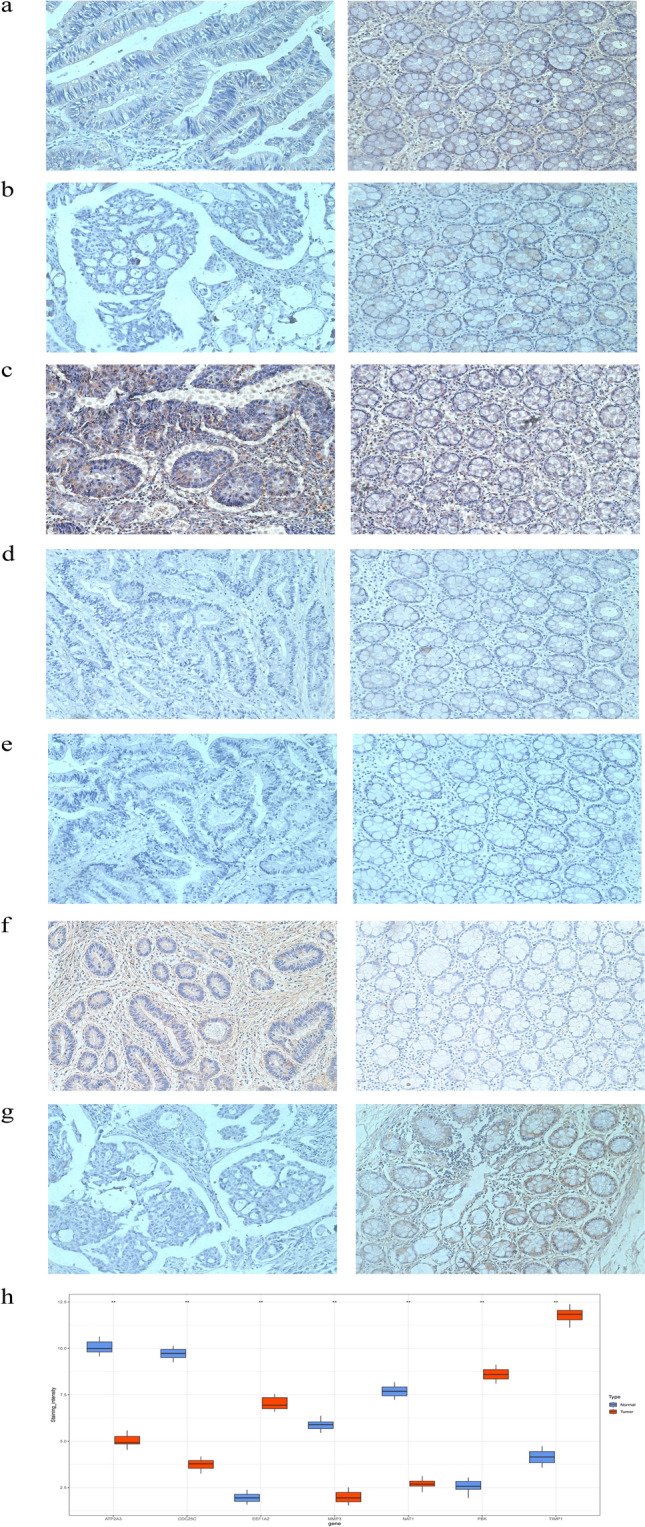
Model genes expression in COAD validated by clinical samples. Representative images and quantification of IHC staining with (**a**) ATP2A3, (**b**) CDC25C, (**c**) EEF1A2, (**d**) TIMP1, (**e**) NAT1, (**f**) PBK, and (**g**) MMP3.Magnification: 20x. p-values were obtained by unpaired *t* test. All data are represented by mean ± SD.

Compared with tumor adjacent tissues (Fig. 10), EEF1A2, PBK and TIMP1 showed significant upregulation in colon adenocarcinoma, while ATP2A3, CDC25C, MMP3 and NAT1 showed significant downregulation. This outcome, is consistent with the results of the risk score model established by us.

## Discussion

Anoikis is a type of programmed cell death that occurs when cells lose their proper extracellular matrix, thereby disrupting integrin attachment^24^. This is an important mechanism to prevent dysplastic cells from proliferating and attaching to inappropriate substrates^25^. Anoikis is also an important body defense that prevents detached cells from attaching to new substrates in the wrong place, thereby preventing their growth and shrinkage^26^. Anoikis is critical for tissue homeostasis and development as it prevents colonization elsewhere by detached epithelial cells^27^. Typically, detachment of normal epithelial cells results in loss of key survival factors and programmed cell death, termed anoikis^28^. Resistance to anoikis, a specific mode of apoptosis activated during cell division, is a critical step in cancer metastasis^29^. Several studies have shown that the initiation of apoptosis depends on both intrinsic and extrinsic signaling pathways^30^. Anoikis can be activated by various intracellular signals, such as DNA damage and endoplasmic reticulum stress, and mitochondria play an important role in the regulation of apoptosis^31^. This impaired function of hypoxia is a hallmark of tumor cells and can lead to tumor invasion and migration, distant metastasis, and drug resistance^32–34^. Anoikis is now of particular interest to the scientific community because anchorage-dependent growth and epithelial-mesenchymal transition, two properties associated with Anoikis resistance, are key steps in tumor progression and dissemination of metastatic cancer cells^35–39^. However, the effects of anoikis-related genes on COAD invasion, migration and drug resistance and their roles in predicting COAD prognosis are rarely studied. Malagobadan S found that the overexpression of this novel miRNA, miR-6744-5p promotes insomnia in Luminal A and triple-negative breast cancer cell lines and directly targets the NAT1 enzyme^40^. Cai Jiaqin &’s research found that the expression of NAT1 was significantly reduced in colorectal cancer, which was independently related to the poor prognosis of colorectal cancer patients. NAT1 can exert anti-tumor activity by inhibiting the phosphorylation of pi3k/Akt/mTOR signaling pathway^41^. Dixie E found that expression of EEF1A2 did not alter sensitivity to anoikis in SK-OV-3 cells^42^. As an epithelial-mesenchymal transition gene, EEF1A2 was found to be strongly associated with the clinical outcome of COAD and has been used as a biomarker and therapeutic drug target in the literature^43,44^. Cui Tang’ research results showed that HOXB13 plays a tumor-promoting role in HCC cells, promotes HCC drug resistance by up-regulating CDC25C, and improves the ability of cells to resist anoikis^45^. These studies found that CDC25C may be an important target gene in patients with COAD because they are related to metabolism, cell cycle and tumor progression^46,47^. Mariana Toricelli found that Timp1 confers cell survival through activation of the PDK1 pathway and that Timp1 and AKT cooperate to confer resistance to anoikis in metastatic melanoma cells^48^. TIMP1 stimulates cell proliferation and has anti-apoptotic functions^49^. In colorectal cancer, upregulation of TIMP1 in tumor tissue compared with normal tissue^50^ is considered to be an independent prognostic factor for disease free survival^51^. MMP3 (Matrix Metallopeptidase 3) is a protein-coding gene, and MMP3-related diseases include Coronary Heart Disease 6 and conjunctivochalasis. Its associated pathways include downstream GPCR signaling and trimerization of collagen chains. Akira Koshino demonstrated that PBK is an immunohistochemical marker of good clinical outcome in CRC patients. PBK-mediated upregulation of cell proliferation and inhibition of CRC cell migration and invasion have also been demonstrated^52^. Overexpression of the PBK gene is associated with tumorigenesis^53^. Immunohistochemical analysis of PBK/TOPK expression can be used as an independent marker for the prognosis of CRC patients^54,55^.ATP2A3 encodes a SERCA pump that pumps Ca^2+^ into the lumen of the ER. One study showed that Serca3 expression is regulated by the proximal ATP2A3 promoter during the induction of epithelial cell differentiation^56^. Previous studies have shown low or no expression of SERCA3 in colon cancer cell lines^57^. Therefore, increased expression of SERCA3 in gastrointestinal cancer may be a prognostic biomarker. Brouland et al. We found that SERCA3 levels were inversely correlated with poorly differentiated epithelial cells. Furthermore, SERCA3 expression is lower in adenocarcinomas^58^. These results suggest a strong correlation between transition from adenomatous polyposis to adenocarcinoma, COAD development, and aberrant SERCA3 expression^59^. Previous studies have shown that these model genes are related to the occurrence and progression of most tumors, which is consistent with our research results.

Here, we demonstrate global genetic alterations associated with anoikis at the gene and transcriptional levels and demonstrate the cross-correlation of COAD. We developed a risk assessment model to predict prognosis and patient response to immunotherapy and targeted therapies. Examining the differential expression of anoikis-related genes in COAD will not only improve our understanding of the aggressiveness of COAD, but will also help in the development of more personalized and accurate immunotherapy regimens. In this experiment, we constructed a prognostic COAD risk assessment model based on the differential expression of anoikis-related genes. We investigated the value of risk scores in predicting the response of COAD to immunotherapy and analyzed the differential expression of immune cells in high-risk and low-risk cancers. The results demonstrate the usefulness of using this risk assessment model to predict patient response to immunotherapy. Examining risk score-based treatment outcomes in COAD patients, we found an interaction between risk score and drug sensitivity. Regulation of apoptosis, signal transduction and metabolism play an active role in the treatment of COAD.

## Conclusion

In this study, we systematically generated and evaluated a COAD risk score based on seven anoikis-related genes. Systematic assessment of risk scores contributes to a better understanding of invasiveness and leads to more individualized and precise treatment strategies. Our model showed good performance in both the training and validation datasets and was further supported by immunohistochemistry.

Our study has profound implications for future research and clinical practice. Our model can also enable the identification of potential therapeutic targets and biomarkers for colon adenocarcinoma. In addition, our model can enable the classification of patients into different risk levels and the customization of treatment options based on their individual prognosis. Our study fosters the progress of precision oncology and personalized medicine for colon adenocarcinoma patients.

### Supplementary Information

Supplementary Figure S1.

Supplementary Figure S2.

Supplementary Figure S3.

Supplementary Legends.

Supplementary Tables.

Supplementary Tables.

## Data Availability

TCGAData Poral: https://portal.gdc.cancer.gov/ (accessed on 15March 2022); GEO Datasets: https://www.ncbi.nlm.nih.gov/gds/ (accessed on 18 December 2022); UCSC Xena: https://xenabrowser.net/ (accessed on 15 December 2022).
