# Supplementary figures and images for: The anoikis-related gene signature predicts survival accurately in colon adenocarcinoma
Source: Sci Rep. 2023 Aug 25;13:13919. doi: 10.1038/s41598-023-40907-x (PMC10457303; doi:10.1038/s41598-023-40907-x)

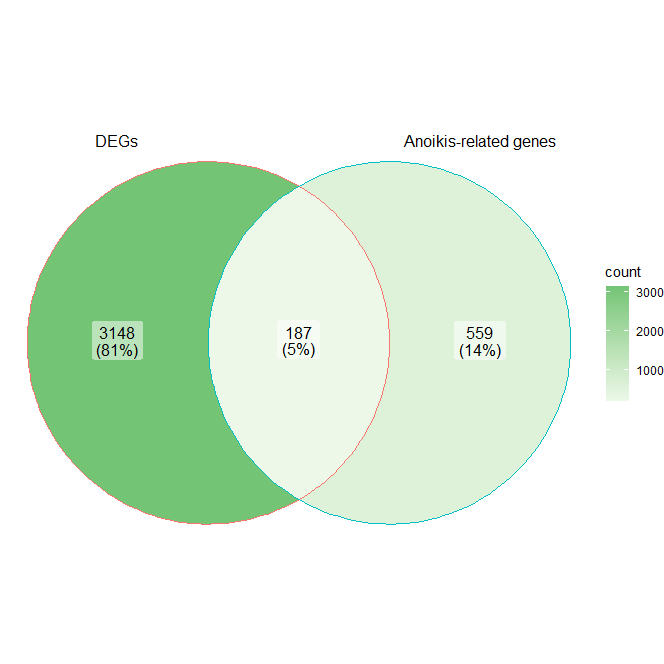

Supplement: Supplementary file 1 — Supplementary Figure S1. [file 41598_2023_40907_MOESM1_ESM.tiff]

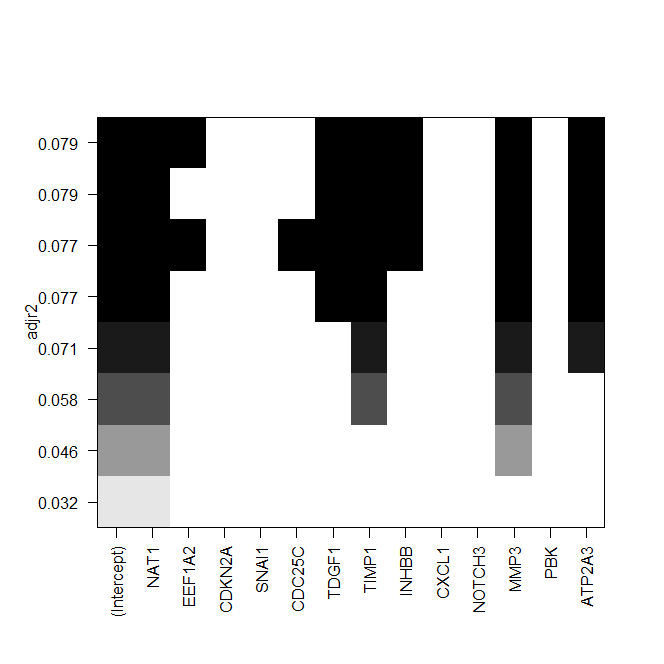

Supplement: Supplementary file 2 — Supplementary Figure S2. [file 41598_2023_40907_MOESM2_ESM.tiff]

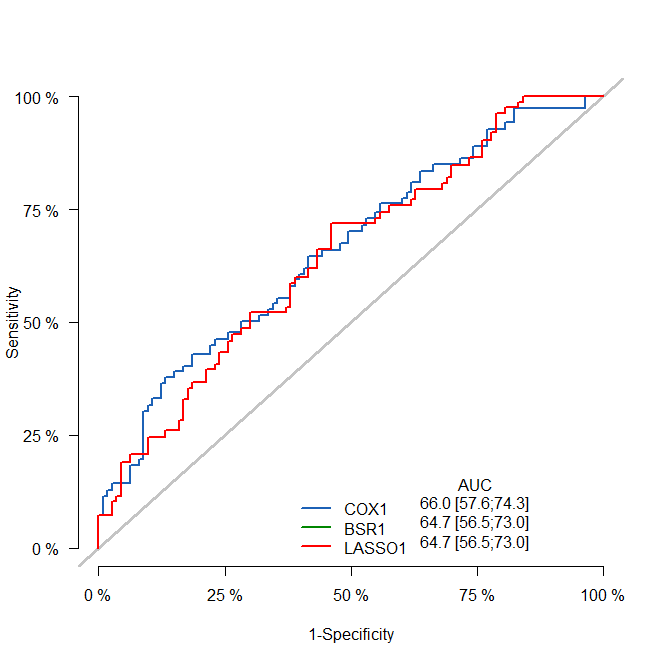

Supplement: Supplementary file 3 — Supplementary Figure S3. [file 41598_2023_40907_MOESM3_ESM.tiff]
