# Supplementary material for: The anoikis-related gene signature predicts survival accurately in colon adenocarcinoma
Source: Sci Rep. 2023 Aug 25;13:13919. doi: 10.1038/s41598-023-40907-x (PMC10457303; doi:10.1038/s41598-023-40907-x)
Supplement: Supplementary file 4 — Supplementary Legends. [file 41598_2023_40907_MOESM4_ESM.docx]

Supplementary Materials:

Figure S1: Venn Diagram of DEGs and anoikis-related genes.;

Figure S2: Results for All-Subsets Regression (BSR) ;

Figure S3: the AUC results for comparing the three methods;

Table S1: Table S1: Results of differential expression of genes in various datasets;

Table S2: GO analysis results of GSEA;

Table S3: KEGG analysis results of GSEA;

Table S4: Results of GO analysis with DEGs；

Table S5:Results of KEGG analysis with DEGs;

Table S6:anoikis-related genes from GeneCards;

Table S7: Intersection of DEGs and anoikis-related genes;

Table S8: OS results for anoikis-related genes(intersection with DEGs) derived from univariate Cox proportional hazards model;

Table S9: PFS results for anoikis-related genes(intersection with DEGs) derived from univariate Cox proportional hazards model;

Table S10: Calculating HR for Risk Score;

Table S11: Calculating C index for Nomogram.
